# A comprehensive analysis of the microbial communities of healthy and diseased marine macroalgae and the detection of known and potential bacterial pathogens

**DOI:** 10.3389/fmicb.2015.00146

**Published:** 2015-02-24

**Authors:** Enrique Zozaya-Valdes, Suhelen Egan, Torsten Thomas

**Affiliations:** Centre for Marine Bio-Innovation, School of Biotechnology and Biomolecular Sciences, The University of New South WalesSydney, NSW, Australia

**Keywords:** delisea pulchra, bacterial disease, macroalgae, bleaching, opportunistic pathogens

## Abstract

Microorganisms are increasingly being recognized as the causative agents in the diseases of marine higher organisms, such as corals, sponges, and macroalgae. *Delisea pulchra* is a common, temperate red macroalga, which suffers from a bleaching disease. Two bacterial strains, *Nautella italica* R11 and *Phaeobacter gallaeciensis* LSS9, have been shown *in vitro* to cause bleaching symptoms, but previous work has failed to detect them during a natural bleaching event. To provide a link between *in vitro* observations and natural occurrences of the disease, we employ here deep-sequencing of the 16S rRNA gene to comprehensively analyze the community composition of healthy and diseased *D. pulchra* samples from two separate locations. We observed operational taxonomic units (OTUs) with 100% identity and coverage to the 16S RNA gene sequence of both *in vitro* pathogens, but only the OTU with similarity to strain LSS9 showed a statistically significant higher abundance in diseased samples. Our analysis also reveals the existence of other bacterial groups within the families *Rhodobacteraceae* and *Flavobacteriaceae* that strongly contribute to difference between diseased and healthy samples and thus these groups potentially contain novel macroalgal pathogens and/or saprophytes. Together our results provide evidence for the ecological relevance of one kind of *in vitro* pathogen, but also highlight the possibility that multiple opportunistic pathogens are involved in the bleaching disease of *D. pulchra*.

## Introduction

Marine sessile macroorganisms, such as seaweed, sponges, and corals, are often colonized by a large number and diversity of bacteria, with which they can have either positive, neutral or negative interactions (Ainsworth et al., 2010; Egan et al., 2012; Hollants et al., 2013). In recent years, disease caused by microorganisms, have been increasingly recognized as a major negative interaction that influences the composition and function of benthic community members (Bourne et al., 2009; Egan et al., 2013). Disease of marine invertebrates and macroalgae has also been clearly linked to changes in the marine environment, such as anthropogenic stressors (pollutants, urbanization etc.) and climate change (e.g., Campbell et al., 2011). However, linking bacterial pathogens to particular diseases in the marine environment is often challenging (Rosenberg et al., 2009; Egan et al., 2013). This can be due to many factors, including the opportunistic nature of pathogens, the existence of multiple pathogens causing the same disease and/or the inability to sensitively detect a particular pathogen in complex environmental samples.

*Delisea pulchra* is a marine red macroalgae commonly found across the temperate Eastern coast of Australia, but is also found as widespread as Japan and Antarctica (Papenfuss, 1964). *D. pulchra* suffers from a bleaching disease, which is characterized by a loss of pigments and which occurs more frequently during summer months, when ultra-violet light radiation and temperature are elevated (Campbell et al., 2011). The bleaching disease has also been shown to have a significant impact on fecundity and survival of the red algae (Campbell et al., 2014). The involvement of bacteria in the bleaching disease is implied by a significant difference in the bacterial community between healthy and diseased *D. pulchra* individuals (Campbell et al., 2011; Fernandes et al., 2012). Furthermore, two pathogens, *Nautella italica* R11 and *Phaeobacter gallaeciensis* LSS9, have been isolated that can cause the bleaching disease *in vitro* (Case et al., 2011; Fernandes et al., 2011). In the laboratory, these two pathogens can invade the tissue of *D. pulchra* under conditions of elevated temperature and when the alga's chemical defense based on UV-sensitive molecules called furanones is reduced. These *in vitro* experiments link pathogen function with the relevant ecological stressors or changes. However, previous studies using denaturing gradient gel electrophoresis (DGGE) from multiple bleaching events (Campbell et al., 2011) and 16S rRNA gene sequencing of clone libraries from a single bleaching event could not detect the two *in vitro* pathogens (Fernandes et al., 2012). Therefore, a link between the *in vitro* bleaching observations (Case et al., 2011; Fernandes et al., 2011) and environmental disease events (Campbell et al., 2014) remains to be determined.

The previous inability of Fernandes et al. (2012) to detect *N. italica* R11 and *P. gallaeciensis* LSS9 *in vivo* may be due to the limited number of samples analyzed and/ or the relatively shallow sequencing analysis of the microbial community of *D. pulchra*. To address this issue, we use here deep-sequencing of the 16S rRNA gene to investigate the microbial community on healthy and bleached individuals from two, natural disease events.

## Results

### Deep 16S rRNA gene sequencing of microbial communities shows OTUs matching *in vitro* pathogens in environmental samples

**Figure 1:**
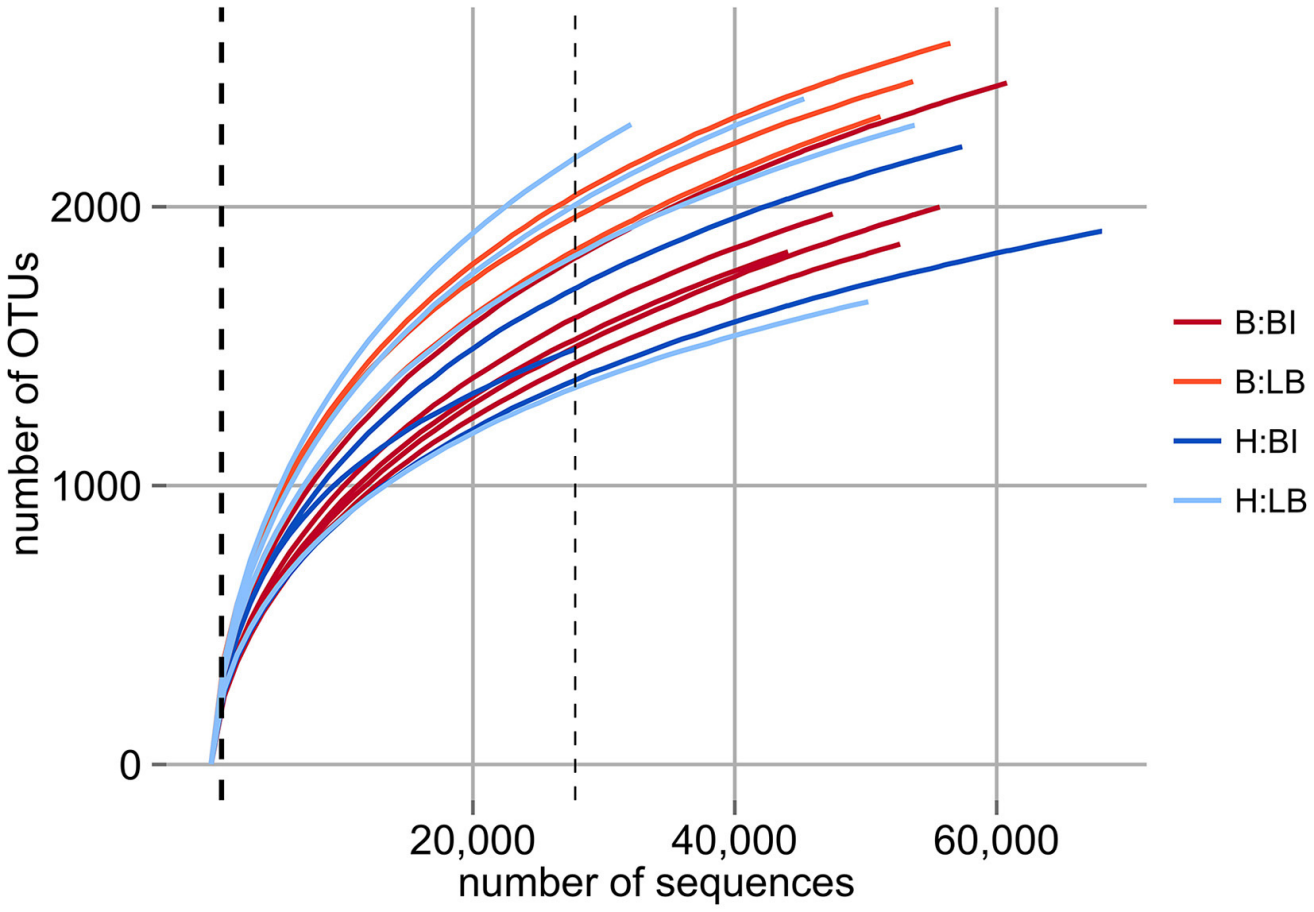
**Rarefaction analysis of *D. pulchra* microbial community samples with OTUs defined at a 16S rRNA V4 region sequence identity of 97%**. Bleached (B) and healthy (H) samples from Bare Island (BI) and Long Bay (LB) are shown. The thick dashed line, which essentially overlaps with the y-axis, shows the sequencing depth of a previous study based on 16S rRNA gene clone libraries (Fernandes et al., 2011). The thin dashed line shows the minimum number of sequences for any sample analyzed here and to which level all samples were subsampled for subsequent community comparison.

In this study we analyzed replicate samples for two separate bleaching events of natural *D. pulchra* populations in two locations (Bare Island and Long Bay) that occurred during the austral summer of 2008 (see Table S1) (Campbell et al., 2011). Using deep-sequencing of the 16S rRNA gene V4 region with the Illumina HiSeq 2000 platform, we obtained 100 bp reads ranging from 64,151, to 142,324 sequences per microbial community sample after stringent quality filtering (see Experimental Procedures and Table S1). These reads were clustered into operational taxonomic units (OTUs) at both 97% and 100% identity. After removal of spurious OTUs and adjusting for the variation in the 16S rRNA gene copy number (see Experimental Procedures) the sequence abundances per sample ranged from 27,815 to 68,051 for the 97% identity OTUs and from 24,995 to 64,048 for 100% identity OTUs. Rarefaction analysis indicated that the sequencing effort started to saturate the diversity of the 16S rRNA gene fragment for all samples (see Figure 1) and this was further supported by Good's coverage estimates of greater than 97% for both OTU definitions (see Table S1). At the level of phylogenetic resolution provided by the 16S rRNA gene fragment sequenced, our results show an almost complete sampling of the bacterial diversity present on the surface of *D. pulchra*, which was not achieved with previous Sanger-based sequencing efforts (Fernandes et al., 2012, see thick, dashed line in Figure 1).

**Figure 2:**
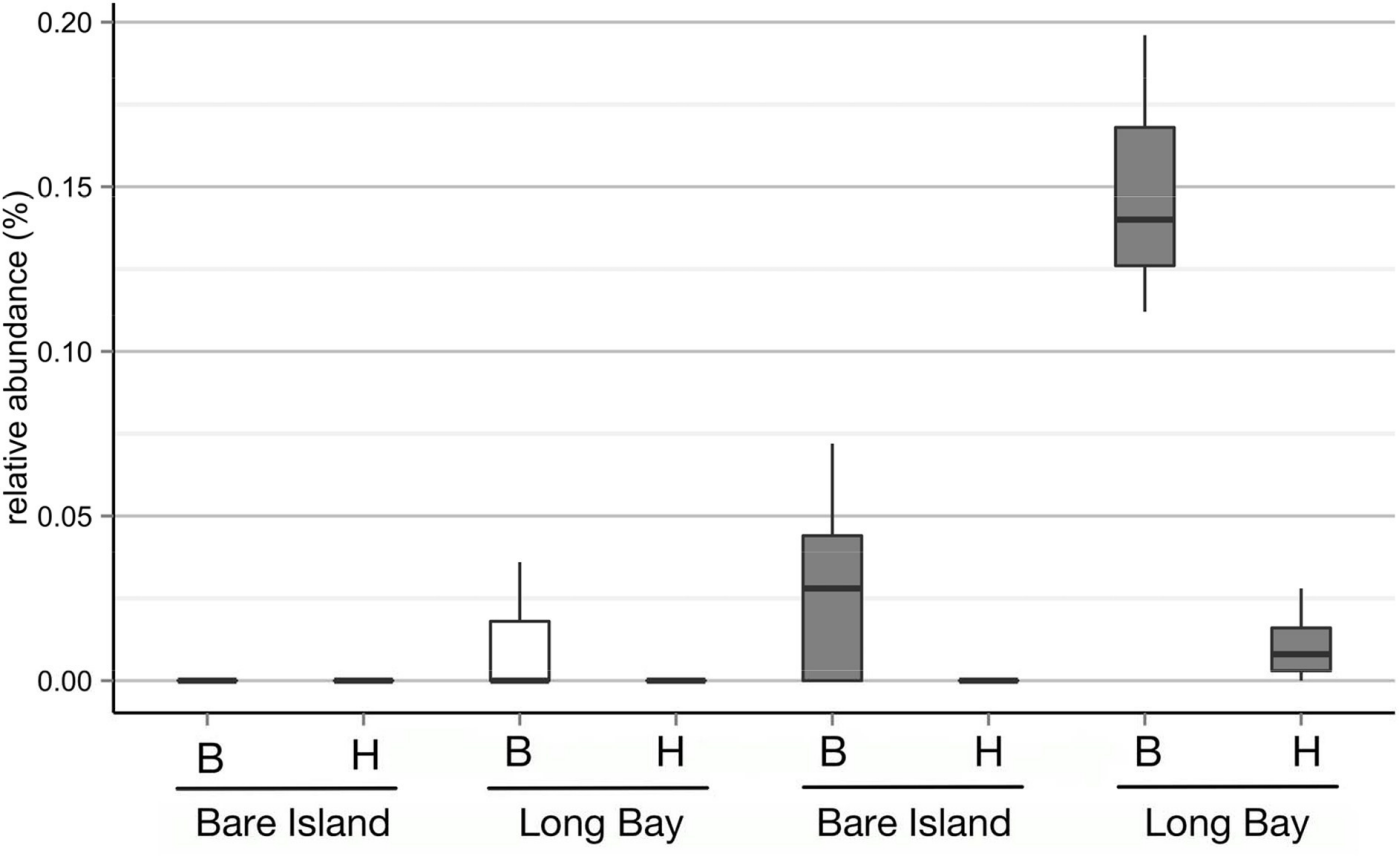
**Relative abundances of OTUs defined at a sequence identity of 100% for the 16S rRNA V4 region and with 100% identity to the 16S rRNA gene of *N. italica* R11 (white boxes) and *P. gallaeciensis* LSS9 (gray boxes) on *D. pulchra* collected at different locations (Bare Island and Long Bay) and with different health status (B, bleached; H, healthy)**.

The extent of the sampling effort should detect OTUs with similarity to the previously described *in vitro* pathogens *N. italica* R11 and *P. gallaeciensis* LSS9, if they are present. In order to match the *in vitro* pathogens to OTUs with the highest phylogenetic resolution that can be obtained with our data, only here we used the OTUs defined at a 100% identity cutoff. Using a 100% query coverage and identity cut-off, a single OTU could be found to match the 16S rRNA gene sequence for each pathogen. These two OTUs occurred with abundances between 0.004% (which is the lowest possible given the sequence depth achieved) and 0.196% (Figure 2). Statistical analysis of the relative abundances in healthy and bleached samples of the two different bleaching events showed that only the OTU that matched to *P. gallaeciensis* LSS9 was significantly more abundant in the bleached samples (*p* = 0.006) overall, and in particular in Long Bay (*p* = 0.012) (Figure 2, Table S2). The putative *N. italica* R11 OTU was only detected in one sample (a bleached individual from Long Bay).

### Difference on bacterial communities of healthy and bleached *D. pulchra*

Given that the two OTUs assigned to the *in vitro* pathogens only made up a small fraction of the overall community, we investigated, if other changes occur in the community that correlated with the bleaching of *D. pulchra*. Based on OTUs defined at a 97% identity cut-off, *D. pulchra* communities had a richness (Chao1) that ranged between 1940 and 3034 and diversity (inverse Simpson index) were between 10.5 and 99.4 (Table S1), but were not significantly different between healthy and bleached samples overall or in either location (Table 1).

**Table 1:** **Hypothesis tests of different measurements for microbial communities of *Delisea pulchra* from Bare Island (BI) and Long Bay (LB)**.

	**Chao**		**InvSimpson**		**Composition**		**Structure**	
	***F***	***p*-value**	***F***	***p*-value**	**Deviance**	***p*-value**	**Deviance**	***p*-value**
Location	4.8	0.05	10.67	0.008^*^	7755	0.029^*^	10145	0.015^*^
Condition	1.89	0.2	0.054	0.82	1220	0.002^*^	16441	0.001^*^
Location × condition	0	0.99	0.06	0.81	3534	0.004^*^	5517	0.014^*^
Condition BI	1.980	0.21	0.19	0.68	6864	0.047^*^	9189	0.022^*^
Condition LB	0.56	0.49	0.0002	0.990	7977	0.043^*^	12239.75	0.029^*^

For richness (Chao) and diversity (InvSimpson) a conventional ANOVA was used, while for composition and structure a MGLM-ANOVA was employed.

* *denotes p-values below significance level of 0.05*.

**Figure 3:**
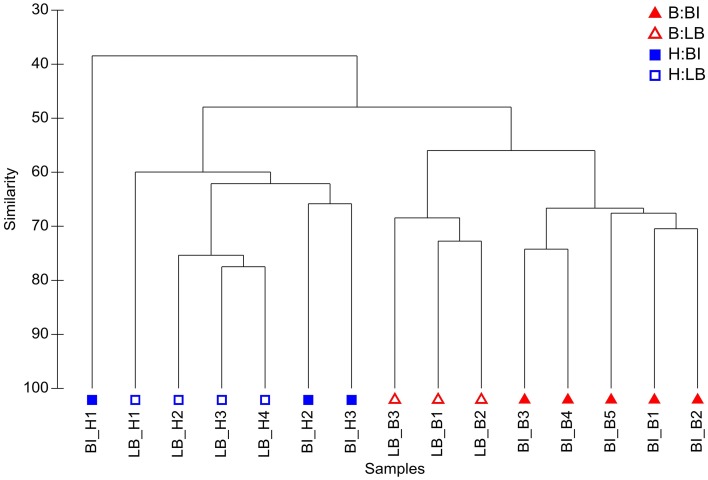
**Hierarchical agglomerative clustering based on Bray Curtis similarity of microbial community structure on *D. pulchra***. Bleached (B) and healthy (H) samples from Bare Island (BI) and Long Bay (LB) are shown. For this analysis OTUs were defined at a sequence identity of 97% for the 16S rRNA V4 region.

In contrast, comparisons of community composition (presence/absence data) and structure (abundance data) using a Bray-Curtis similarity revealed clear differences between healthy and bleached algae (see Figures 3, S1). Hypothesis testing based on a multivariate generalized linear model (MGLM) analysis of variance (MGLM-ANOVA) showed a significant interaction between condition and location (Table 1). This indicates that the magnitude and/or direction of the differences between microbial communities of healthy and bleached samples can be affected by the location of the samples. Therefore, further comparisons were made within each sampling site to appropriately assess whether there are significant differences between healthy and bleached samples. Comparisons within each site showed significant difference in the community composition and structure between healthy and bleached samples from both sampling sites (Table 1), which is consistent with observations made in two previous studies (Campbell et al., 2011; Fernandes et al., 2012).

**Figure 4:**
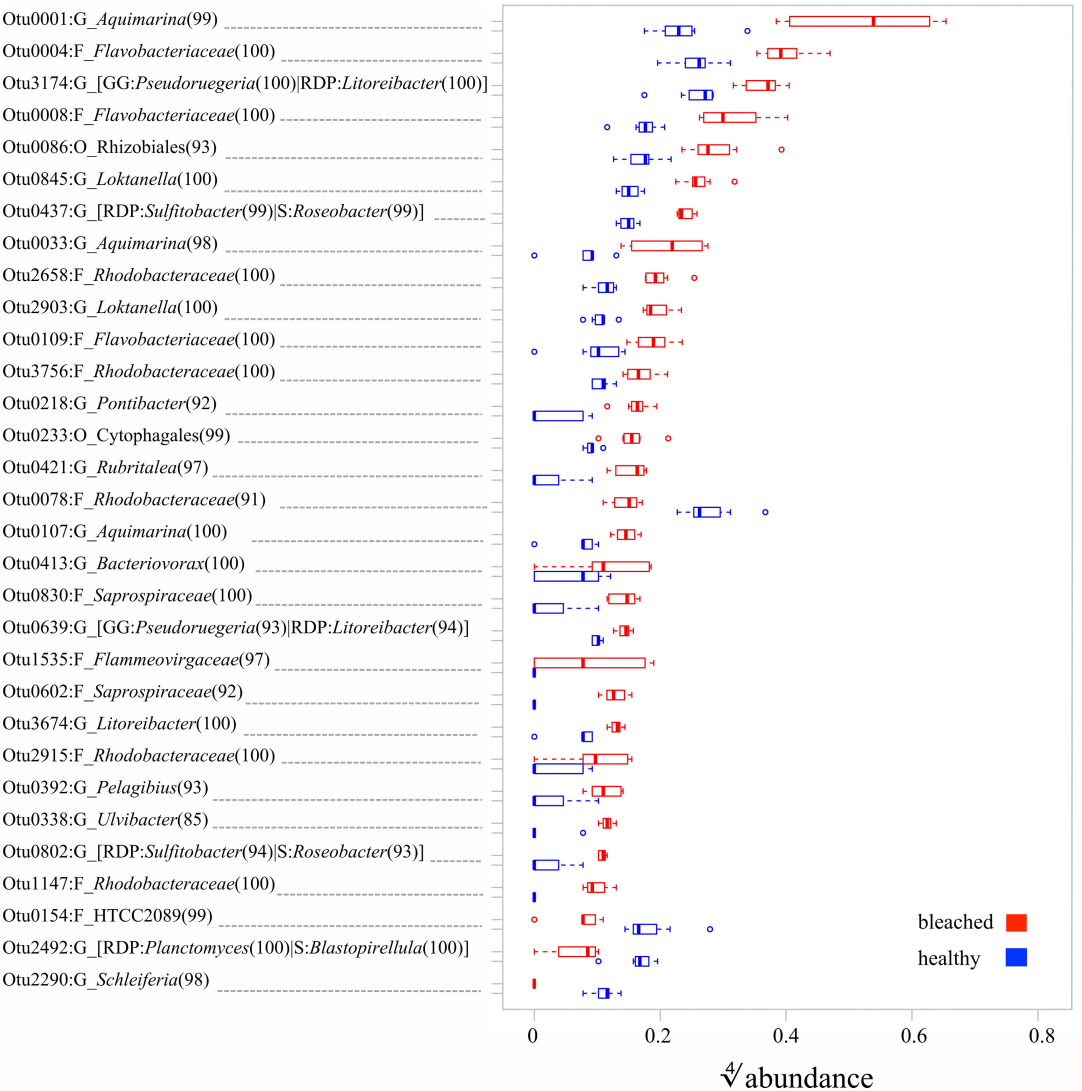
**Relative abundances of OTUs (defined at a sequence identity of 97% for the 16S rRNA V4 region) with statistically significant (MGLM-ANOVA with *p*_adjusted_**< 0.05**) difference between healthy and bleached *D. pulchra* samples**. On the y-axis, the letter after the colon indicates the taxonomic level, at which the OTU could be classified (O, Order; F, Family; G, Genus). Alternative taxonomic assignment by different databases (RDP, Ribosomal Database Project; GG, Greengenes; S, Silva) are shown in square brackets. The consensus confidence of the OTU classifications are shown in round brackets. The OTUs are ordered in decreasing order from the top by their relative abundance in bleached samples.

Having established general differences in the communities, we next asked which OTUs contribute in a statistically significant way to the observed changes. Univariate (i.e., OTU-by-OTU) results of the MGLM-ANOVA test using a conservative adjustment for multiple testing, showed that 31 OTUs are statistically significant different between healthy and bleached samples across the two bleaching events (Figure 4, Table S3). These OTUs, which represent 0.8% of all OTUs, contribute to the top 5% of differences (as measured by the deviance test statistic) between healthy and bleached samples and 27 of these OTUs were more abundant in bleached samples. These abundant OTUs in bleached samples had a wide taxonomic distribution, belonging to three different phyla, six classes, and nine families (Figure 4). At the phylum level, the majority of the OTUs belong to the Proteobacteria (52%) and Bacteroidetes (44%), with one OTU assigned to the Verrucomicrobia (4%). At the lowest taxonomy level, at which still most of the OTUs could be classified (93% at family level), these OTUs are classified as *Rhodobacteraceae* (41%) and *Flavobacteriaceae* (26%). The remaining OTUs belong to the *Rhodospirillaceae* (4%), *Bacteriovoracaceae* (4%), *Saprospiraceae* (7%), *Cytophagaceae* (4%), *Flammeovirgaceae* (4%), and *Verrumicrobiaceae* (4%). The four OTUs that were more abundant in healthy samples can be assigned to the family *Rhodobacteraceae*, the genera *Schleiferia* (phylum Flavobacteria), *Planctomyces* or *Blastopirellula* (as classified with RDP (Cole et al., 2014) or SILVA (Quast et al., 2013), respectively), and the class Gammaproteobacteria.

## Discussion

### The presence of *in vitro* pathogens on *D. pulchra* and their contribution to changes in the microbial community of diseased individuals

Detection of known pathogens in natural disease events is often limited by sensitivity i.e., the lack of appropriate sequencing depth resulting in incomplete description of the microbial diversity present in a sample. A comprehensive analysis of microbial communities can be achieved by deep-sequencing of part of the 16S rRNA gene with current sequencing technologies (Caporaso et al., 2012; Werner et al., 2012), however as with any PCR-based technology bias due to primer binding and amplification efficiencies can potentially misrepresent the relative abundance of certain microbial groups (e.g., Engelbrektson et al., 2010).

After the complete sequence processing, which includes OTU clustering at 100% identity, removal of potentially spurious OTUs and 16S rRNA gene copy number correction, we obtained an average of 46,255 sequence reads per sample (or a 60 times increase to the per sample sequence average of previous study by Fernandes et al., 2011), which resulted in an estimated coverage of the community diversity of greater than 97% (with the caveats of bias, mentioned above) and a detection limited for any given OTU at a relative abundance of 0.004%.

This sequence analysis allowed us to detect an OTU with similarity to *N. italica* R11 only in one bleached sample and not in any other of the 14 samples analyzed. Considering the high sampling coverage and the low detection limit reached, this would suggest that *N. italica* R11 is unlikely to play a major role in the bleaching events observed here and that it constitutes a rare member of the microbial community of the *D. pulchra* individuals analysed here.

An OTU matching to *P. gallaeciensis* LSS9 was detected in 9 out of 15 samples (six bleached samples and three healthy ones) and this OTU showed a statistically significant higher abundance in bleached samples in general and in Long Bay when assessing the difference within sites (Table S2). The limited phylogenetic resolution of the V4 region used here however did not allow us to unambiguously assign the OTU to strain LSS9 as BLAST analysis also showed 100% identity to 16S rRNA genes from other members of the *Rhodobacteraceae* family. Nevertheless, the statistically significant increase of the OTU in one bleaching event would indicate that *P. gallaeciensis* LSS9 or closely-related organisms might contribute to the disease in natural settings.

The inability to consistently detect higher relative abundance for the OTUs matching to either *in vitro* pathogen on bleached samples could be due to two alternative, but not mutually exclusive scenarios. Firstly, the bleaching disease of *D. pulchra* could progress through multiple stages, in which different sets of pathogens colonize and proliferate at any one time. In this scenario, the inability to consistently detect *N. italica* R11 or *P. gallaeciensis* LSS9 could be simply because they were not abundant in the disease stages in which the samples were taken. A well-understood example for “staged diseases” is given by the Black Band Disease of corals, which involves distinct morphological stages (e.g., cyanobacterial patches and microbial lesions) and defined microbial succession patterns (Sato et al., 2013). Further temporal studies of the bleaching disease of *D. pulchra* would be required to define potential disease stages, however field observations have so far failed to observe distinct morphological states prior to bleaching.

The second scenario is that there is a pool of pathogens naturally present on healthy *D. pulchra* living as commensals. When the natural host defenses are compromised due to detrimental environmental factors (e.g., UV stress etc.), then these pathogens proliferate in an opportunistic fashion (Egan et al., 2012; Fernandes et al., 2012). Under this scenario, each time a bleaching event occurs, a different subset of opportunists dominate based on chance, similar to what has been postulated for the colonization and proliferation of planktonic bacteria on the surface of the green alga *Ulva australis* (Burke et al., 2011). Support for the second scenario is given by the fact that the majority (80%) of OTUs (defined at a 97% identity cut-off) that contribute most to the overall community difference between healthy and bleached samples (top 20% of the deviance) are in fact different in each bleaching event. Moreover, the majority of OTUs that are abundant on diseased samples were also present in healthy samples (71% and 88% of the OTUs that contributed most to the difference in Bare Island and Long Bay, respectively), which would be consistent with their commensal role in healthy macroalgae.

### Detection of multiple potential pathogens of *D. pulchra*

In addition of attempting to detect *in vitro* pathogens, we also investigated if and which other OTUs (97% identity cut-off) make a significant contribution to the differences between microbial communities of bleached and healthy *D. pulchra* (Figure 4). Most OTUs with significant difference in relative abundance where enriched in bleached samples and of those the majority were classified as belonging to the families *Rhodobacteraceae* (41%) and *Flavobacteriaceae* (26%).

Within the *Rhodobacteraceae*, all OTUs that could be classified to the genus level (7 out of 11) belong to the Roseobacter clade. The Roseobacter clade is one of nine major marine clades (Giovannoni and Rappe, 2000) and its members have been found in practically every marine niche, including in associations with marine eukaryotes, such as corals, sponges, cephalopods, scallop larvae, seagrasses as well as micro- and macroalgae (Buchan et al., 2005; Wagner-Döbler and Biebl, 2006). Roseobacter clade bacteria are in fact frequently isolated from macroalgae (Brinkhoff et al., 2008) and have the capacity to utilize algal osmolytes, like putrescine, taurine, creatine, sarcosine, and dimethylsulfoniopropionate (DMSP) (Wagner-Döbler and Biebl, 2006; Kalhoefer et al., 2011; Thole et al., 2012). Of particular interest is DMSP, as several marine micro- and macroalage contain it in high concentrations (Yoch, 2002). Therefore, degrading tissue of bleached *D. pulchra* could possibly be a source of DMSP (and other osmolytes) for saprophytic behavior of members of the Roseobacter clade.

Alternatively, the Roseobacter-clade OTUs enriched on bleached samples could constitute opportunistic pathogens, with similar properties to *N. italica* R11 and *P. gallaeciensis* LSS9, which also belong to this group. The Roseobacter clade contains many other known or putative pathogens, such as *Roseovarius crassostreae*, which causes the Roseovarius Oyster Disease (Maloy et al., 2007); the strains that are consistently detected in corals affected with white plague-like disease and black band disease (Cooney et al., 2002; Pantos et al., 2003); and diseased individuals of the sponge *Rhopaloides odorabile* (Webster et al., 2002). As for algal disease, the bacterium *Ruegeria atlantica* has been shown *in vitro* to synthesize compounds that lyse the dinoflagellate *Alexandrium catenella* (Amaro et al., 2005) and Roseobacter strains can cause a tumor-like gall disease in the red alga *Prionitis lanceolata* (Ashen and Goff, 1998).

Within the *Flavobacteriaceae*, 3 out of 7 OTUs were assigned to the genus *Aquimarina*, with one of them (OTU1) representing the most abundant OTU in the community of bleached *D. pulchra* (relative abundance of 9.3%; see in Figure 4). Members of the *Aquimarina* have previously been reported to be associated with diseases of marine eukaryotes. For example, *Aquimarina homaria* dominates (jointly with another *Flavobacteriaceae* species) the microbial communities associated with shell lesions in American lobster (Chistoserdov et al., 2012; Quinn et al., 2012). In addition, *Aquimarina agaralytic*, which was isolated from a red macroalga, was found to posses a large number of diverse agarases (Lin et al., 2012a, b) that may function to degrade host tissue, and *Aquimarina salinaria* was shown to produce metabolites with algicidal activity (Chen et al., 2011). Other OTUs that contributed to the difference in bleached *D. pulchra* were assigned to the order Cytophagales, the family *Saprospiraceae*, and the genus *Saprospira*. These taxa along with the *Flavobacteriaceae* OTUs, belong to the Cytophaga/Flavobacterium/Bacteroidetes (CFB) group, which contains most of the algicidal bacteria isolated from marine and coastal environments (Fandino et al., 2001; Furusawa et al., 2003; Mayali and Azam, 2004; Roth et al., 2008; Chen et al., 2011). The potential role of such algicidal bacteria as macroalgal pathogens is thus worth further investigation. Additionally, members of the CFB group have been show to contribute to secondary infections of red alga *Chondrus crispus* after initial infection by an endophytic green algae (Correa and McLachlan, 1994; Craigie and Correa, 1996; Goecke et al., 2010).

In conclusions, the contribution that specific OTUs of the *Rhodobacteraceae* and *Flavobacteriaceae*/CFB make to the community differences between healthy and diseased samples, together with the fact that members of these groups have previously been implicated in marine diseases, suggests that these groups of organisms contain prime candidates for alternative pathogens of *D. pulchra*. While the environmental observation made here shows a correlation with disease, isolates from these groups need to be obtained in the future in order to clearly demonstrate them as causative agents in either *in vitro* or *in vivo* settings. If more strains with pathogenic properties can be demonstrated to exist, then this would further support the model that environmental diseases, such as the bleaching disease of *D. pulchra*, result from the action and activities of widespread opportunistic pathogens (Egan et al., 2013). How these potential pathogens respond to environmental conditions and interact with other community members is likely important for the development of disease. Complex shifts in the microbial community have also been observed in diseases affecting other marine organisms, such as corals (Rosenberg et al., 2007; Thurber et al., 2009; Ainsworth et al., 2010; Mouchka et al., 2010; Littman et al., 2011) and sponges (Webster et al., 2008; Angermeier et al., 2011; Fan et al., 2013; Olson et al., 2014), and for those diseases a model involving multiple pathogens might also be applicable.

## Experimental procedures

### Sampling and microbial community DNA isolation

Replicate samples of healthy (*n* = 7) and bleached (*n* = 8) *D. pulchra* individuals were collected by SCUBA at depths of around 9 m at Bare Island (S33°59′30.80″, E151°13′53.60″) and Long Bay (S 33°57′59.79″, E151°15′26.11″), off the coast of Sydney, Australia, during the austral summer (12 February 2008) (see Table S1). Each algal sample was enclosed individually in clip-sealed plastic bags *in situ* and transported to the laboratory, where they were rinsed in filtered seawater three times to remove any loosely associated epibionts. Algae were then gently patted with sterile paper tissue to remove excess seawater and then freeze-dried. DNA was extracted from freeze-dried algal samples (50–100 mg) using a ZR Soil Microbe DNA extraction kit (Zymo) following the manufacturer's protocol as previously described (Campbell et al., 2011).

### 16S rRNA gene sequencing and processing

DNA samples were processed through the Earth Microbiome Project (EMP) (Gilbert et al., 2010) to generate amplicons for the variable region 4 (V4) of the 16S rRNA gene using the universal bacterial/archaeal primers 515F/806R. The amplified samples were sequenced on a HiSeq Illumina 2000 platform with 100 bps from the 515F primer. Reads were demultiplexed and quality trimmed using the QIIME software. For a more detailed description of these methods see www.earthmicrobiome.org. The sequencing data have been deposited in the Short Reads Archive under accession SRX824554 (Bare Island) and SRX824555 (Long Bay).

Sequences were further processed in Mothur (Schloss et al., 2009) using the guidelines of the MiSeq standard operational procedure (http://www.mothur.org/wiki/MiSeq_SOP) with the following modifications: sequences were trimmed with parameters qwindowaverage = 30, qwindowsize = 5, maxambig = 0, maxhomop = 8, minlength = 100. The alignment was done using the reference alignment of Silva release 102 cut to the V4 region of the 16S rRNA gene. Aligned sequences were pre-clustered using diffs = 1. Chimeras were removed using chimera.uchime with dereplicate = t and contaminants (i.e., sequences from chloroplasts, mitochondria, eukaryotes or with unknown taxonomical affiliation) were filtered after classifying the sequences with the RDP version 9 reference taxonomy. Sequences were clustered into OTUs at 97% or 100% using the cluster.split command (splitmethod = classify, taxlevel = 4) and the same reference as with the alignment.

### OTU-based community analysis

Due to the large number of reads produced by the HiSeq Illumina platform, sequences that have been filtered for high quality can still produce spurious OTUs. However, an additional abundance-based OTU filtering can produce an OTU collection that better reflects the true diversity of the microbial sample (Bokulich et al., 2013). Here, OTUs were removed that had an absolute abundance across samples lower than 15 (or relative abundance of 0.0009% or lower across samples), which is the total number of samples analyzed here.

The number of reads per OTU was further corrected for the known or inferred 16S rRNA gene copy number of the taxon that the OTU was assigned to. For this, the Greengenes database (version of October 2012) (DeSantis et al., 2006) was downloaded and its taxonomy file clustered at 0.99 identity were formatted according to Mothur specifications. This reference dataset was used in Mothur to classify sequences and obtain a majority consensus taxonomy using the classify.otu command with default settings. This taxonomic classification was used to create a QIIME-formatted OTU table, which was then employed in Copyrighter (Angly et al., 2014) using the default trait estimates file (ssu_img40_gg201210.txt) and option –t. The OTU table with corrected absolute abundances was converted back into the Mothur format and used for all subsequent analysis. After this adjustment, the minimum relative abundance of any OTU in any sample was 0.004%.

The number of OTUs, coverage, Chao1 and the Inverse Simpson diversity index were calculated with Mothur for 1000 random subsamples using the smallest 16S rRNA gene abundance in any sample (i.e., sample BI_H1 with an abundance after 16S rRNA gene copy number correction of 27,815). The average of the subsamples were used to perform Analysis of Variance (ANOVA) tests between samples types. Bray-Curtis similarities were calculated for the communities' composition (presence/absence data) and structure (abundance data) and hierarchical agglomerative clustering dendograms of samples were generated using Primer-E v6 (Clarke and Gorley, 2006). To calculate the Bray-Curtis similarity of community structure, the OTU abundances were square-root transformed. To test whether there were statistically significant differences between healthy and bleached samples in both composition and structure a two-factor design (Location with levels “Bare Island” and “Long Bay” and Condition with levels “healthy” and “bleached”) was used to adjust the data to a MGLM using the mvabund package (Wang et al., 2012). In this approach, each OTU is treated as a variable that is fitted to a separate generalized linear model (GLM) using a negative binomial distribution for the analysis of community structure and a binomial distribution for composition analysis. For multivariate hypothesis testing, the ANOVA function (which implements an analysis of deviance) was applied to the MGLM using the p.uni argument set to return univariate OTU-by-OTU results adjusted to control the family-wise error rate across OTUs. Additionally, these univariate ANOVA-like tests were ordered by deviance to identify the OTUs that contribute more strongly to the overall difference between healthy and bleached samples.

Using the databases Silva (release 119), RDP (PDS version 10) and Greengenes (release of August 2013) in Mothur, three separate majority-based consensus taxonomic classifications (classify.otu command with default parameters) were obtained for the OTUs that had a statistically significant effect by Condition. A consensus of the three classifications was manually built by reporting only the deepest taxonomic assignment, using the highest consensus confidence results observed and showing alternative taxa when different classifications were obtained with the different reference database.

### Definition of OTUs that match the 16S rRNA gene of known, *in vitro* pathogens

To investigate the presence of the strains *N. italica* R11 and *P. gallaeciensis* LSS9 their 16S rRNA gene were searched against the 100% OTU clusters using blastn and a cutoff for coverage and identify of 100% for the OTU sequence. Using this criteria one OTU cluster was found for each pathogen. Because the OTU cluster assigned to *N. italica* R11 was present in only one sample, no further analysis were done on it. For the OTU assigned to *P. gallaeciensis* LSS9, the mvabund software package (Wang et al., 2012) was used to fit the by-sample standardized OTU abundance to a GLM using again a negative binomial distribution. This GLM was then used to test the significance of the difference between healthy and bleached samples using the ANOVA function (analysis of deviance).

### Conflict of interest statement

The authors declare that the research was conducted in the absence of any commercial or financial relationships that could be construed as a potential conflict of interest.

## References

[B1] Ainsworth T. D. Thurber R. V. Gates R. D. (2010). The future of coral reefs: a microbial perspective. Trends Ecol. Evol. 25, 233–240. 10.1016/j.tree.2009.11.001 20006405

[B2] Amaro A. M. Fuentes M. S. Ogalde S. R. Venegas J. A. Suárez-Isla B. A. (2005). Identification and characterization of potentially algal-lytic marine bacteria strongly associated with the toxic dinoflagellate *Alexandrium catenella*. J. Eukaryot. Microbiol. 52, 191–200. 10.1111/j.1550-7408.2005.00031.x 15926994

[B3] Angermeier H. Kamke J. Abdelmohsen U. R. Krohne G. Pawlik J. R. Lindquist N. L.. (2011). The pathology of sponge orange band disease affecting the Caribbean barrel sponge *Xestospongia muta*. FEMS Microbiol. Ecol. 75, 218–230. 10.1111/j.1574-6941.2010.01001.x 21118276

[B4] Angly F. E. Dennis P. G. Skarshewski A. Vanwonterghem I. Hugenholtz P. Tyson G. W. (2014). CopyRighter: a rapid tool for improving the accuracy of microbial community profiles through lineage-specific gene copy number correction. Microbiome 2:11. 10.1186/2049-2618-2-11 24708850 PMC4021573

[B5] Ashen J. B. Goff L. J. (1998). Galls on the marine red alga *Prionitis lanceolata* (*Halymeniaceae*): specific induction and subsequentdevelopment of an algal–bacterial symbiosis. Am. J. Bot. 185, 1710–1721. 10.2307/2446505 21719414

[B6] Bokulich N. A. Subramanian S. Faith J. J. Gevers D. Gordon J. I. Knight R.. (2013). Quality-filtering vastly improves diversity estimates from Illumina amplicon sequencing. Nat. Methods 10, 57–59. 10.1038/nmeth.2276 23202435 PMC3531572

[B7] Bourne D. G. Garren M. Work T. M. Rosenberg E. Smith G. W. Harvell C. D. (2009). Microbial disease and the coral holobiont. Trends Microbiol. 17, 554–562. 10.1016/j.tim.2009.09.004 19822428

[B8] Brinkhoff T. Giebel H.-A. Simon M. (2008). Diversity, ecology, and genomics of the Roseobacter clade: a short overview. Arch. Microbiol. 189, 531–539. 10.1007/s00203-008-0353-y 18253713

[B9] Buchan A. González J. M. Moran M. A. (2005). Overview of the marine roseobacter lineage. Appl. Environ. Microbiol. 71, 5665–5677. 10.1128/AEM.71.10.5665-5677.2005 16204474 PMC1265941

[B10] Burke C. Steinberg P. Rusch D. Kjelleberg S. Thomas T. (2011). Bacterial community assembly based on functional genes rather than species. Proc. Natl. Acad. Sci. U.S.A. 108, 14288–14293. 10.1073/pnas.1101591108 21825123 PMC3161577

[B11] Campbell A. H. Harder T. Nielsen S. Kjelleberg S. Steinberg P. D. (2011). Climate change and disease: bleaching of a chemically defended seaweed. Glob. Change Biol. 17, 2958–2970 10.1111/j.1365-2486.2011.02456.x

[B12] Campbell A. H. Vergés A. Steinberg P. D. (2014). Demographic consequences of disease in a habitat-forming seaweed and impacts on interactions between natural enemies. Ecology 95, 142–152. 10.1890/13-0213.1 24649654

[B13] Caporaso J. G. Lauber C. L. Walters W. A. Berg-Lyons D. Huntley J. Fierer N.. (2012). Ultra-high-throughput microbial community analysis on the Illumina HiSeq and MiSeq platforms. ISME J. 6, 1621–1624. 10.1038/ismej.2012.8 22402401 PMC3400413

[B14] Case R. J. Longford S. R. Campbell A. H. Low A. Tujula N. Steinberg P. D.. (2011). Temperature induced bacterial virulence and bleaching disease in a chemically defended marine macroalga. Environ. Microbiol. 13, 529–537. 10.1111/j.1462-2920.2010.02356.x 20946533

[B15] Chen W.-M. Sheu F.-S. Sheu S.-Y. (2011). Aquimarina salinaria *sp. nov*., a novel algicidal bacterium isolated from a saltpan. Arch. Microbiol. 194, 103–112. 10.1007/s00203-011-0730-9 21766186

[B16] Chistoserdov A. Y. Quinn R. A. Gubbala S. L. Smolowitz R. (2012). Bacterial communities associated with lesions of shell disease in the american lobster (*Homarus americanus*, Milne-Edwards. J. Shellfish Res. 31, 449–462. 10.2983/035.031.020523750952

[B17] Clarke K. R. Gorley R. N. (2006). PRIMER v6: User Manual/Tutorial. Plymouth: PRIMER-E.

[B18] Cole J. R. Wang Q. Fish J. A. Chai B. McGarrell D. M. Sun Y.. (2014). Ribosomal database project: data and tools for high throughput rRNA analysis. Nucleic Acids Res. 42, D633–D642. 10.1093/nar/gkt1244 24288368 PMC3965039

[B19] Cooney R. P. Pantos O. Le Tissier M. D. A. Barer M. R. O'Donnell A. G. Bythell J. C. (2002). Characterization of the bacterial consortium associated with black band disease in coral using molecular microbiological techniques. Environ. Microbiol. 4, 401–413. 10.1046/j.1462-2920.2002.00308.x 12123476

[B20] Correa J. A. McLachlan J. L. (1994). Endophytic algae of *Chondrus crispus* (Rhodophyta). V. Fine structure of the infection by Acrochaete operculata (Chlorophyta). Eur. J. Phycol. 29, 33–47 10.1080/09670269400650461

[B21] Craigie J. S. Correa J. A. (1996). Etiology of infectious diseases in cultivated *Chondrus crispus* (Gigartinales, Rhodophyta), in Fifteenth International Seaweed Symposium, eds Lindstrom S. C. Chapman D. 1. (Dordrecht: Springer Netherlands), 97–104.

[B22] DeSantis T. Z. Hugenholtz P. Larsen N. Rojas M. Brodie E. L. Keller K.. (2006). Greengenes, a chimera-checked 16S rRNA gene database and workbench compatible with ARB. Appl. Environ. Microbiol. 72, 5069–5072. 10.1128/AEM.03006-05 16820507 PMC1489311

[B23] Egan S. Fernandes N. D. Kumar V. Gardiner M. Thomas T. (2013). Bacterial pathogens, virulence mechanism and host defence in marine macroalgae. Environ. Microbiol. 16, 925–938. 10.1111/1462-2920.12288 24112830

[B24] Egan S. Harder T. Burke C. Steinberg P. Kjelleberg S. Thomas T. (2012). The seaweed holobiont: understanding seaweed-bacteria interactions. FEMS Microbiol. Rev. 37, 462–476 10.1111/1574-6976.12011 23157386

[B25] Engelbrektson A. Kunin V. Wrighton K. C. Zvenigorodsky N. Chen F. Ochman H.. (2010). Experimental factors affecting PCR-based estimates of microbial species richness and evenness. ISME J. 4, 642–647. 10.1038/ismej.2009.153 20090784

[B26] Fan L. Liu M. Simister R. Webster N. S. Thomas T. (2013). Marine microbial symbiosis heats up: the phylogenetic and functional response of a sponge holobiont to thermal stress. ISME J. 7, 991–1002. 10.1038/ismej.2012.165 23283017 PMC3635241

[B27] Fandino L. B. Riemann L. Steward G. F. Long R. A. Azam F. (2001). Variations in bacterial community structure during a dinoflagellate bloom analyzed by DGGE and 16S rDNA sequencing. Aquat. Microb. Ecol. 23:119 10.3354/ame023119

[B28] Fernandes N. Case R. J. Longford S. R. Seyedsayamdost M. R. Steinberg P. D. Kjelleberg S.. (2011). Genomes and virulence factors of novel bacterial pathogens causing bleaching disease in the marine red alga *Delisea pulchra*. PLoS ONE 6:e27387. 10.1371/journal.pone.0027387 22162749 PMC3230580

[B29] Fernandes N. Steinberg P. Rusch D. Kjelleberg S. Thomas T. (2012). Community structure and functional gene profile of bacteria on healthy and diseased thalli of the red seaweed *Delisea pulchra*. PLoS ONE 7:e50854. 10.1371/journal.pone.0050854 23226544 PMC3513314

[B30] Furusawa G. Yoshikawa T. Yasuda A. Sakata T. (2003). Algicidal activity and gliding motility of *Saprospira* sp. SS98-5. Can. J. Microbiol. 49, 92–100. 10.1139/w03-017 12718397

[B31] Gilbert J. A. Meyer F. Antonopoulos D. Balaji P. Brown C. T. Brown C. T.. (2010). Meeting report: the terabase metagenomics workshop and the vision of an Earth microbiome project. Stand. Genomic Sci. 3, 243–248. 10.4056/sigs.1433550 21304727 PMC3035311

[B32] Giovannoni S. J. Rappe M. (2000). Evolution, diversity, and molecular ecology of marine prokaryotes, in Microbial Ecology of the Oceans, ed Kirchman D. L. (New York, NY: John Wiley & Sons, Inc), 47–84.

[B33] Goecke F. Labes A. Wiese J. Imhoff J. (2010). Chemical interactions between marine macroalgae and bacteria. Mar. Ecol. Prog. Ser. 409, 267–299 10.3354/meps08607

[B34] Hollants J. Leliaert F. De Clerck O. Willems A. (2013). What we can learn from sushi: a review on seaweed-bacterial associations. FEMS Microbiol. Ecol. 83, 1–16. 10.1111/j.1574-6941.2012.01446.x 22775757

[B35] Kalhoefer D. Thole S. Voget S. Lehmann R. Liesegang H. Wollher A. (2011). Comparative genome analysis and genome-guided physiological analysis of *Roseobacter litoralis*. BMC Genomics 12:324 10.1186/1471-2164-12-324 21693016 PMC3141670

[B36] Lin B. Lu G. Li S. Hu Z. Chen H. (2012a). Draft genome sequence of the novel agarolytic bacterium *Aquimarina agarilytica* ZC1. J. Bacteriol. 194, 2769. 10.1128/JB.00311-12 22535944 PMC3347208

[B37] Lin B. Lu G. Zheng Y. Xie W. Li S. Hu Z. (2012b). Aquimarina agarilytica *sp. nov.*, an agarolytic species isolated from a red alga. Int. J. Syst. Evol. Microbiol. 62, 869–873. 10.1099/ijs.0.027136-0 21622833

[B38] Littman R. Willis B. L. Bourne D. G. (2011). Metagenomic analysis of the coral holobiont during a natural bleaching event on the Great Barrier Reef. Environ. Microbiol. Rep. 3, 651–660. 10.1111/j.1758-2229.2010.00234.x 23761353

[B39] Maloy A. P. Ford S. E. Karney R. C. Boettcher K. J. (2007). *Roseovarius crassostreae*, the etiological agent of Juvenile Oyster Disease (now to be known as Roseovarius Oyster Disease) in Crassostrea virginica. Aquaculture 269, 71–83 10.1016/j.aquaculture.2007.04.008

[B40] Mayali X. Azam F. (2004). Algicidal bacteria in the sea and their impact on algal blooms. J. Eukaryot. Microbiol. 51, 139–144. 10.1111/j.1550-7408.2004.tb00538.x 15134248

[B41] Mouchka M. E. Hewson I. Harvell C. D. (2010). Coral-associated bacterial assemblages: current knowledge and the potential for climate-driven impacts. Integr. Comp. Biol. 50, 662–674. 10.1093/icb/icq061 21558231

[B42] Olson J. B. Thacker R. W. Gochfeld D. J. (2014). Molecular community profiling reveals impacts of time, space, and disease status on the bacterial community associated with the Caribbean sponge *Aplysina cauliformis*. FEMS Microbiol. Ecol. 87, 268–279. 10.1111/1574-6941.12222 24112035

[B43] Pantos O. Cooney R. P. Le Tissier M. D. A. Barer M. R. O'Donnell A. G. Bythell J. C. (2003). The bacterial ecology of a plague-like disease affecting the Caribbean coral *Montastrea annularis*. Environ. Microbiol. 5, 370–382. 10.1046/j.1462-2920.2003.00427.x 12713463

[B44] Papenfuss G. (1964). Catalogue and bibliography of Antarctic and sub-Antarctic benthic marine algae, in Bibliography of the Antarctic Seas, ed Lee M. O. (Washington DC: American Geophysical Union), 1–76.

[B45] Quast C. Pruesse E. Yilmaz P. Gerken J. Schweer T. Yarza P.. (2013). The SILVA ribosomal RNA gene database project: improved data processing and web-based tools. Nucleic Acids Res. 41, D590–D596. 10.1093/nar/gks1219 23193283 PMC3531112

[B46] Quinn R. A. Metzler A. Smolowitz R. M. Tlusty M. Chistoserdov A. Y. (2012). Exposures of homarus americanusshell to three bacteria isolated from naturally occurring epizootic shell disease lesions. J. Shellfish Res. 31, 485–493 10.2983/035.031.0208

[B47] Rosenberg E. Koren O. Reshef L. Efrony R. Zilber-Rosenberg I. (2007). The role of microorganisms in coral health, disease and evolution. Nat. Rev. Microbiol. 5, 355–362. 10.1038/nrmicro1635 17384666

[B48] Rosenberg E. Kushmaro A. Kramarsky-Winter E. Banin E. Yossi L. (2009). The role of microorganisms in coral bleaching. ISME J. 3, 139–146. 10.1038/ismej.2008.104 19005495

[B49] Roth P. B. Twiner M. J. Mikulski C. M. Barnhorst A. B. Doucette G. J. (2008). Comparative analysis of two algicidal bacteria active against the red tide dinoflagellate *Karenia brevis*. Harmful Algae 7, 682–691 10.1016/j.hal.2008.02.002

[B50] Sato Y. Willis B. L. Bourne D. G. (2013). Pyrosequencing-based profiling of archaeal and bacterial 16S rRNA genes identifies a novel archaeon associated with black band disease in corals. Environ. Microbiol. 15, 2994–3007. 10.1111/1462-2920 24112537

[B51] Schloss P. D. Westcott S. L. Ryabin T. Hall J. R. Hartmann M. Hollister E. B.. (2009). Introducing mothur: open-source, platform-independent, community-supported software for describing and comparing microbial communities. Appl. Environ. Microbiol. 75, 7537–7541. 10.1128/AEM.01541-09 19801464 PMC2786419

[B52] Thole S. Kalhoefer D. Voget S. Berger M. Engelhardt T. Liesegang H.. (2012). *Phaeobacter gallaeciensis* genomes from globally opposite locations reveal high similarity of adaptation to surface life. ISME J. 6, 2229–2244. 10.1038/ismej.2012.62 22717884 PMC3504968

[B53] Thurber R. V. Willner-Hall D. Rodriguez-Mueller B. Desnues C. Edwards R. A. Angly F.. (2009). Metagenomic analysis of stressed coral holobionts. Environ. Microbiol. 11, 2148–2163. 10.1111/j.1462-2920.2009.01935.x 19397678

[B54] Wagner-Döbler I. Biebl H. (2006). Environmental biology of the marine Roseobacter lineage. Annu. Rev. Microbiol. 60, 255–280. 10.1146/annurev.micro.60.080805.142115 16719716

[B55] Wang Y. Naumann U. Wright S. T. Warton D. I. (2012). mvabund- an Rpackage for model-based analysis of multivariate abundance data. Methods Ecol. Evol. 3, 471–474. 10.1111/j.2041-210X.2012.00190.x19432789

[B56] Webster N. S. Negri A. P. Webb R. I. Hill R. T. (2002). A spongin-boring a-proteobacterium is the etiological agent of disease in the Great Barrier Reef sponge *Rhopaloeides odorabile*. Mar. Ecol. Prog. Ser. 232, 305–309 10.3354/meps232305

[B57] Webster N. S. Xavier J. R. Freckelton M. Motti C. A. Cobb R. (2008). Shifts in microbial and chemical patterns within the marine sponge *Aplysina aerophoba* during a disease outbreak. Environ. Microbiol. 10, 3366–3376. 10.1111/j.1462-2920.2008.01734.x 18783385

[B58] Werner J. J. Zhou D. Caporaso J. G. Knight R. Angenent L. T. (2012). Comparison of Illumina paired-end and single-direction sequencing for microbial 16S rRNA gene amplicon surveys. ISME J. 6, 1273–1276. 10.1038/ismej.2011.186 22170427 PMC3379627

[B59] Yoch D. C. (2002). Dimethylsulfoniopropionate: its sources, role in the marine food web, and biological degradation to dimethylsulfide. Appl. Environ. Microbiol. 68, 5804–5815. 10.1128/AEM.68.12.5804-5815.2002 12450799 PMC134419

