# Supplementary material for: A comprehensive analysis of the microbial communities of healthy and diseased marine macroalgae and the detection of known and potential bacterial pathogens
Source: Front Microbiol. 2015 Feb 24;6:146. doi: 10.3389/fmicb.2015.00146 (PMC4338804; doi:10.3389/fmicb.2015.00146)

**Supplementary Material**

**A comprehensive analysis of the microbial communities of healthy and diseased marine macroalgae detects known and potential bacterial pathogens**

Enrique Zozaya Valdes, Suhelen Egan, Torsten Thomas*

Centre for Marine Bio-Innovation and School of Biotechnology and Biomolecular Sciences, The University of New South Wales, Sydney, NSW 2052, Australia

***Correspondence:** Torsten Thomas, Centre for Marine Bio-Innovation, The University of New South Wales, Sydney, NSW 2052, Australia email: [t.thomas@unsw.edu.au](mailto:t.thomas@unsw.edu.au), telephone: +61-2-93853467

**Table S1.** *D. pulchra* epibiotic microbial community samples used in present study and diversity measurements. The data in the table was calculated after quality processing of sequences and after correcting OTU abundances for the variation in16S rRNA gene copy number. The diversity measurements (excepting the number of sequences) were calculated for a 1000 random subsamples at the size of the smallest 16S rRNA gene abundance in any sample. Presented here is average of the 1000 subsamples (see Methods). BI:Bare Island, LB:Long, Bay, B:Bleached, H:Healthy

**Table S2.** Analysis of variance based on a GLM fitted to the relative abundance of an OTU defined at a 100% identity cutoff that matched the 16S rRNA V4 region of *Phaeobacter gallaeciensis* LSS9. * denotes p-values below a significance level of 0.05.

|  | **Deviance** | **p-value** |
| --- | --- | --- |
| **location** | 1.919 | 0.138 |
| **condition** | 11.327 | 0.006* |
| **location X condition** | 1.964 | 0.232 |
| **condition BI** | 4.7 | 0.108 |
| **condition LB** | 11.9 | 0.012* |

**Table S3.** Univariate results of the OTUs that showed a statistically significant effect for condition (healthy vs bleached) in the MGLM-ANOVA test. P-values were adjusted to control for family-wise error rate across OTUs. OTUs were ordered decreasingly by the condition factor’s deviance.

| **OTU ID** | **Location**  **deviance** | **Location**  **p-value** | **Condition**  **deviance** | **Condition**  **p-value** | **LocationXCondition deviance** | **LocationXCondition**  **p-value** |
| --- | --- | --- | --- | --- | --- | --- |
| Otu0437 | 0.995 | 1 | 41.780 | 0.012 | 2.775 | 1 |
| Otu0845 | 0.093 | 1 | 31.529 | 0.024 | 1.815 | 1 |
| Otu0830 | 4.497 | 0.985 | 31.389 | 0.024 | 3.341 | 1 |
| Otu0218 | 0.065 | 1 | 30.484 | 0.025 | 0.045 | 1 |
| Otu0602 | 0.913 | 1 | 28.993 | 0.028 | 0.000 | 1 |
| Otu0639 | 0.001 | 1 | 28.685 | 0.029 | 0.000 | 1 |
| Otu0338 | 0.970 | 1 | 28.498 | 0.029 | 1.389 | 1 |
| Otu2903 | 0.092 | 1 | 28.469 | 0.029 | 0.167 | 1 |
| Otu0078 | 0.367 | 1 | 28.148 | 0.029 | 4.449 | 0.999 |
| Otu2290 | 2.581 | 1 | 27.509 | 0.029 | 0.000 | 1 |
| Otu2658 | 0.389 | 1 | 26.925 | 0.031 | 5.300 | 0.994 |
| Otu0107 | 2.754 | 0.998 | 26.642 | 0.031 | 1.382 | 1 |
| Otu0086 | 1.797 | 1 | 26.375 | 0.032 | 1.574 | 1 |
| Otu3674 | 0.747 | 1 | 25.684 | 0.035 | 0.417 | 1 |
| Otu0001 | 7.249 | 0.865 | 24.798 | 0.04 | 2.630 | 1 |
| Otu0109 | 1.142 | 1 | 24.609 | 0.042 | 0.626 | 1 |
| Otu2492 | 2.956 | 0.997 | 24.250 | 0.043 | 0.232 | 1 |
| Otu2915 | 6.528 | 0.904 | 23.910 | 0.043 | 0.554 | 1 |
| Otu3174 | 0.079 | 1 | 23.709 | 0.044 | 2.248 | 1 |
| Otu0413 | 8.407 | 0.762 | 23.604 | 0.045 | 0.114 | 1 |
| Otu0004 | 0.571 | 1 | 23.514 | 0.045 | 0.227 | 1 |
| Otu1147 | 0.841 | 1 | 23.455 | 0.045 | 0.000 | 1 |
| Otu0421 | 0.036 | 1 | 23.173 | 0.045 | 5.100 | 0.995 |
| Otu0802 | 0.309 | 1 | 22.925 | 0.047 | 0.054 | 1 |
| Otu1535 | 5.112 | 0.967 | 22.901 | 0.047 | 0.000 | 1 |
| Otu0033 | 8.183 | 0.78 | 22.528 | 0.047 | 4.921 | 0.997 |
| Otu3756 | 0.066 | 1 | 22.525 | 0.047 | 0.093 | 1 |
| Otu0392 | 3.051 | 0.997 | 22.444 | 0.048 | 1.664 | 1 |
| Otu0008 | 3.925 | 0.99 | 22.439 | 0.048 | 3.005 | 1 |
| Otu0154 | 0.474 | 1 | 22.425 | 0.048 | 2.074 | 1 |
| Otu0233 | 0.469 | 1 | 22.345 | 0.049 | 0.011 | 1 |

**Figure S1.** Bray-Curtis based hierarchical agglomerative clustering of *D. pulchra* microbial community composition. Bleached (B) and healthy (H) samples from Bare Island (BI) and Long Bay (LB) are shown.


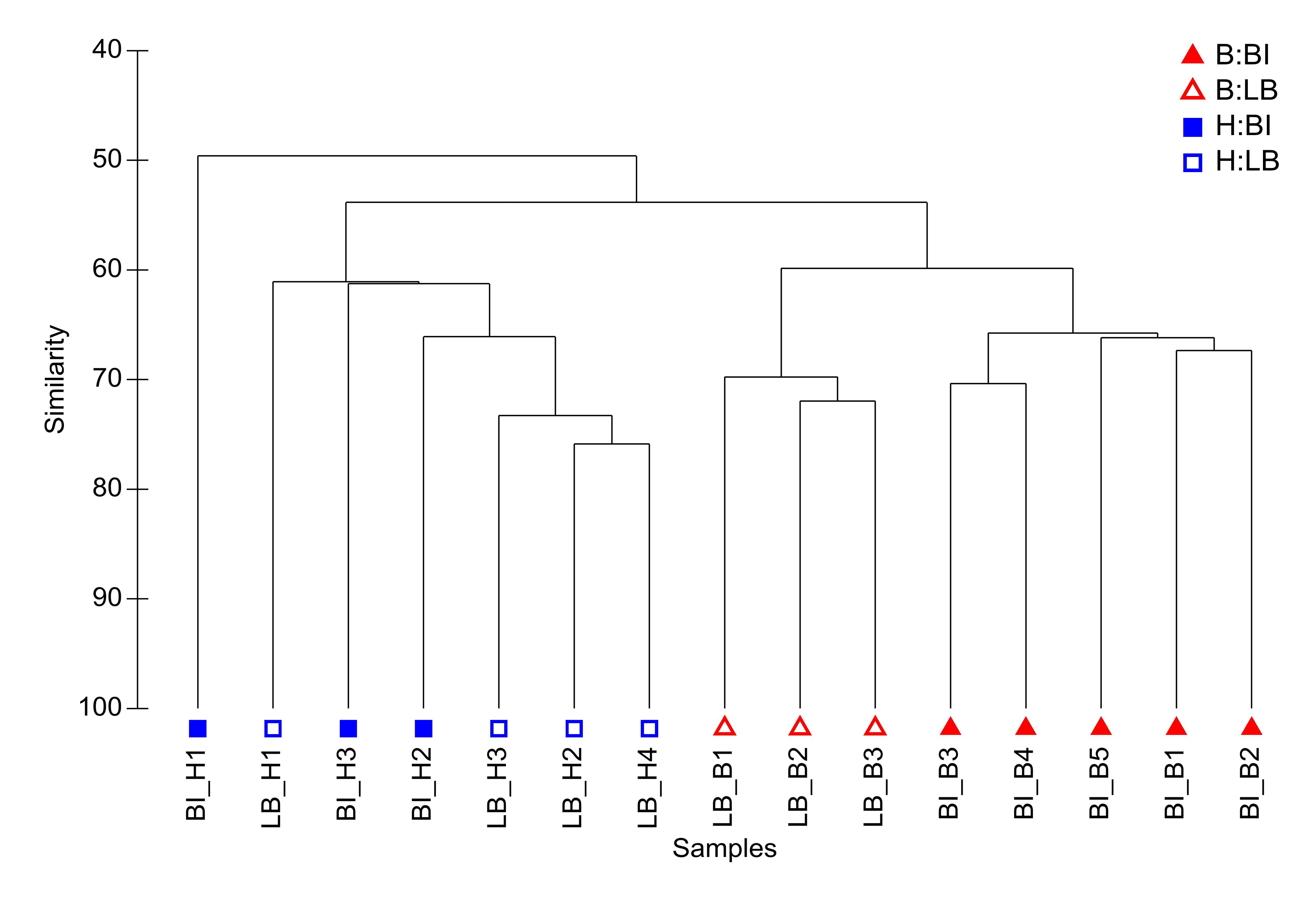

Supplement: Supplementary file 1 [file Supplementary_Materials.docx]
